# Effect of *Moringa oleifera* leaves on hematological profile of fluorosis affected rats

**DOI:** 10.6026/97320630018014

**Published:** 2022-01-31

**Authors:** Pravallika Pagadala, MS Vinutha Shankar, A Hemalatha, KN Shashidhar

**Affiliations:** 1 Department of Physiology, Sri Devaraj Urs Academy of higher education & Research, Tamaka Kolar, Karnataka, 563103, India; 2 Department of Pathology, Sri Devaraj Urs Academy of higher education & Research, Tamaka Kolar, Karnataka, 563103, India; 3 Department of Biochemistry, Sri Devaraj Urs Academy of higher education & Research, Tamaka Kolar, Karnataka, 563103, India

**Keywords:** Fluoride, anemia, intoxification, premature erythrocytes death, drinking water, Moringa Olifera

## Abstract

Fluorosis is a metabolic disease that is endemic in nearly 25 countries with India being one of the most affected. It primarily affects the bone and the teeth. Moringa oleifera (MO) leaves are known to reduce the effect of fluorosis on various tissues. Therefore, it is of interest to document the effect of Moringa oleifera leaves on the hematological profile of fluorosis affected rats. Twenty four Sprague Dawley rats were housed two per cage in a room with 12 hours light and 12 hours dark cycle. The rats were allowed to adjust to the laboratory environment for about one to two weeks before the beginning of the study. This study reveals that MO leaves is effective in reducing the plasma fluoride content. It also helps in improving the Hb % and RBC count in fluorosis affected rats. Data shows that Moringa olifera leaves powder is effective in reducing the plasma fluoride content. It also helps in improving the Hemoglobin percentage & Red Blood Cell count in fluorosis affected rats.

## Background:

Fluoride is known to act as a double edged sword and is the 13th most abundant highly reactive, electronegative halogen with an atomic number 9 in the earth's crust [1-2]. It helps to prevent the formation of dental caries at ingestion in low concentration (<1 ppm) but ingestion in high concentration leads to dental and skeletal lesions, commonly called as fluorosis [3]. There are 25 countries in Asia and Africa which are affected with fluorosis and India is the front manner with a highest prevalence. Present Indian statistics depicts that around 25 million people were affected by fluorosis and 66 million in future including children of age 14 years are at risk of developing fluorosis [4]. Studies show that 39% of population in Karnataka exhibited skeletal fluorosis [4]. Kolar located in the eastern most Karnataka has been reported to have 26,000 people suffering from dental and skeletal fluorosis [4]. Most common causes for fluorosis are through internal route and the main fluoride content is the drinking water. Other sources include exposure to fluoride rich effluents, dust and smoke from aluminum smelters plants, copper, glass, iron, super phosphate fertilizers plants and brick kilns areas [5-6]. The control of fluoride drinking-water is critical in preventing fluorosis. Removal of excessive fluoride from drinking-water is difficult and expensive. Fluorosis also leads to muscle fatigue, muscle weakness, hypothyroidism, anemia, oxidative stress that promotes atherosclerosis & myocardial cell damage, lung parenchymal inflammation, decreased GFR and diabetes mellitus [2]. Anemia has several complications in children and adults especially in pregnant women. During fluorosis anemia is caused due to reduced erythropoietin activity [2]. Some studies showed that fluoride toxicity may increase phagocytic activity of macrophages to engulf more RBC in spleen which contributes to anemia and increased white blood cells causing hematological alterations in rabbits [7]. A study reported that fluoride intoxification may lead to anemia by early hemolysis [8]. Most of these manifestations are, no doubt, nonspecific, but their occurrence in subjects living in fluorosis-endemic areas should alert suspicion. These early warning signs have been extremely helpful in early detection of large numbers of cases in rural areas; prompt intervention programmes (i.e. providing safe drinking water) in these cases have provided considerable relief within a short span of time [9]. Several inorganic and organic treatment methods such as reverse osmosis, nano filtration, electrodialysis, donan dialysis, ultra filtration, ion exchange and adsorption were tried to reduce fluorosis [10-11]. Some studies found that beneficial effect of aqueous extract of Moringa seeds & dried leaf powder to minimise fluoride toxicity in rabbits and calves [12]. Moringa oleifera (MO) leaves belongs to Moringaceae family commonly known as "The Miracle Tree", "Horseradish-tree" or "Ben oil tree" [13]. It is a multipurpose crop, widely cultivated in Africa and Southern Asia and has medicinal and nutritional properties [14,15]. Leaves of the MO tree are noted for high crude protein, energy and appreciable levels of carotene, ascorbic acid, iron, methionine and cysteine with negligible amounts of tannins [16]. Earlier studies have found MO is nontoxic and recommended for therapeutic use in developing countries [17]. Therefore, it is of interest to document the effect of MO leaves on haematological profile of fluorosis affected rats.

## Materials and methods:

### Design of study:

Prospective case control study.

### Number of animals:

Twenty four male Sprague Dawley (SD) rats are included for the study and were categorized into four groups (Group I, Group II, Group III & Group IV) of six animals each which was approved by institutional animal ethics committee (IAEC/PHARMA/SDUMC/2017-18/10a). Group I (n = 6): Control animals had free access to RO water for a period of 30days (reference range of fluoride is 0.3-0.5mg/litre) [18]. Group II (n=6): Sodium fluoride was administered ad libitum in the drinking water at a concentration of 50mg/kg body weight for a period of 30 days [19,22] . Group III (n = 6): This group animals received 50mg/kg of body weight of fluoride in drinking water, supplemented with 200mg/kg of MO orally by mixing with water through an oral gavage bent needle for a period of 30 days [21]. Group IV (n = 6): Animals with continuous access of food and RO water supplemented with MO by mixing with water through a oral gavage bent needle as a vehicle for a period of 30 days.

### Study Location:

This study was done in the department of physiology, Sri Devaraj Urs Medical College. SD rat's 10-12 weeks old, weighing 180-220 gms, were housed two per cage in a room with 12 hour's light & 12 hours dark cycle. The rats were allowed to adjust to the laboratory environment for around one to two weeks before the start of the study. A standard animal feed and drinking water were provided 'ad-libitum'.

### Plant materials:

Semi ripen leaves of M O were washed with clean water to remove dirt and soil. The leaves which are having any outer observable lesions or decayed ones were discarded. The leaves were dried at 60°С upto a constant mass. These dried materials further processed into powder form by passing through grinder, it was kept in air tight sachets till further use [1]. Blood sample were collected by retro orbital puncture from all the groups for complete blood picture & fluoride levels estimation. Plasma was separated from the EDTA blood samples for the estimation of fluoride.

### Complete blood picture & blood smear analysis:

After collecting the EDTA blood samples from all the groups, haemoglobin (Hb) (g/dL), Red blood cell count (RBC), Total leukocyte count (TLC), Packed cell volume (PCV), Differential leukocyte count (DLC) & Reticulocyte count were estimated by automated haematology system analyzer method; peripheral blood smear was studied to know the morphology of cells. Erythrocyte indices such as Mean corpuscular volume (MCV), Mean corpuscular Haemoglobin (MCH), Mean Corpuscular Haemoglobin concentration (MCHC) were calculated.

### Estimation of fluoride:

The fluoride concentration of plasma samples were measured by Ion selective electrode method. This method was adopted by Cernik et al. with modifications of orien model [1].

### Method:

Fluoride Ion Selective electrode (ISE).

### Principle:

The sensing element, epoxy body in Fluoride electrode senses the Fluoride ion containing solution which is in contact with electrode. The strength of electrode potential developed across the sensing element depends on concentration of Fluoride ion (F¯) in solution. The potential developed is measured by pH/mV metre or ISE meter, the value corresponds to the level of F¯ in solution. Confirmation of slope as per user manual is completed. F¯ concentration is expressed in ppm or mole per litre or any convenient unit. Different concentrations of standards are prepared by using the formula: C1 x V1 = C2 x V2. Detection limit: 0.02 ppm.

### Analysis & Statistical Methods:

Data was coded and entered into Microsoft excel data sheet. Quantitative data was represented as mean, confidence interval and categorical data by percentages. Data was analyzed by using two-way analysis of variance (ANOVA) & post hoc analysis to compare between the groups. A p value of less than or equal to 0.05 is considered as statistically significant.

## Results & Discussion:

The results were analyzed using the licensed version of SPSS statistics 20, Mean ± SD was calculated.

## Comparison of findings with Group I & Group II:

RBC count and Hb% were significantly decreased (p<0.05) in Group II as compared with Group I. Fluoride content was increased in Group II compared with Group I and was statistically significant. Parameters such as MCV, MCHC, MCH, TLC, PLT & Rets% were decreased in Group II compared to Group I, but the decrease was not statistically significant.

## Comparison of findings with Group I & Group III:

Parameters such as RBC, Hb, MCV, MCHC & Rets % were within biological reference range in both Group III & Group I. MCH, TLC, PLT and fluoride levels were decreased in Group III compared with Group I. There was no significant difference in parameters between Group I & Group III. Comparison of findings with Group I & Group IV: There was no significant difference in parameters between Group I & Group IV.

## Comparison of findings with Group II and Group III:

RBC count and Hb% were significantly increased in Group III compared with Group II. Similarly Fluoride content was significantly decreased in Group III compared with Group II. There was no significant difference in other parameters such as MCV, MCHC, MCH, TLC, PLT & Rets %.

## Comparison of findings with Group II and Group IV:

RBC count and Hb % were significantly increased in Group IV compared with Group II. Fluoride content was significantly decreased in Group IV when compared with Group II. There was no significant difference in other parameters such as MCV, MCHC, MCH, TLC, PLT & Rets %. MO is a local, natural, cost wise affordable, palatable and abundantly available plant which can be consumed either in the form of leaves or as a vegetable. It is commonly used as a vegetable with many add on advantages. It is a multipurpose crop, widely cultivated in Africa and Southern Asia with medicinal and nutritional properties [14,15]. MO leaves are noted for high levels of crude protein, energy and noticeable levels of carotene, ascorbic acid, iron, methionine and cysteine with insignificant amounts of tannins which may help in ameriolating the toxic effects of fluoride [16]. Its nutritional importance is due to high content of calcium and phosphorus. The biological properties and medicinal functions of MO extracts have been mainly supported by in vitro assays based on their antioxidant capacity and bioactive profile [22,23, 24]. Due to their abundance of phenoilc acids and flavanoids MO leaf extract exhibits antioxidant activity. Studies have demonstrated that the methanolic leaf extract of MO has more chemical constituents than the seed [25]. Studies that have investigated the chemical constituents of the methanolic extract of MO leaves and seeds by using Gas chromatography-mass spectrometry have identified sixteen chemical constituents in the leaf methanolic extract [26]. Low molecular weight water soluble proteins in MO has strong positive charge which attracts highly electronegative fluoride ions resulting in formation of flocculants.

Studies shows that flourosis is known to cause anemia [1,7]. The results of present study has revealed that there was significantly (p<0.05) decrease in the RBC count, Hb% & increase in plasma fluoride content in fluorosis affected rats. This reduction was not seen in MO leaves with fluoride supplemented group compared to controls. Though all the haematological parameters were reduced in Flourosis group as compared with Controls, only Hb% and RBC count were statistically significant. Accumulation of Fluoride takes place in erythrocyte (red blood cells) membrane, which sequentially looses calcium content. The membrane which is lacking in calcium content is flexible and is thrown into folds. The shape of erythrocytes is changed. Such RBCs are called echinocytes, which will be found in circulation. The echinocytes are eliminated from circulation by means of phagocytosis. This would lead to low haemoglobin levels in fluoride toxicity [27]. Some studies suggests that dietary supplement of MO may have the potential of reversing anemia within a short period of administration, because it has been known to contain alkaloids, flavonoids, phytosterols and saponin which are identified to have hemapoietic property. Apart from these bioactive substances in the leaves of MO, it has also been said to be an outstanding source of vitamin A, B, C, minerals like iron as well as protein, which may all contribute to its observed effects on red blood cells [28].

Maryam et al. in their study showed that administration of fluoride orally to rabbits leads to reduction in RBC count, leukocytopenia, monocytosis, eosinopenia, neutrophilia and thrombocytosis and stated that fluoride toxicity may increase phagocytic activity of macrophages to engulf more RBC in spleen which contributes to anemia causing haematological alterations [7]. The present study findings are consistent with the data documented by Maryam et al. and Mandal et al. [1, 7]. We could observe that in fluoride supplemented group significantly reduced was observed only in Hb% and RBC count but not with other blood parameters. Studies conducted by Susheela et al. reported that fluoride intoxification leads to anemia by premature erythrocyte deaths [9]. Peripheral smear examination reveals normocytic normochromic anemia. Mandal et al. have showed that the calves reared in flourotic zone had decreased Hb, PCV, TLC and increased fluoride content supplementation of dried MO fruit powder to those calves resulted in significant reduction in fluoride levels and increase in Hb%, PCV, TLC. They showed that Supplementation of MO fruit powder was able to reduce the plasma fluoride level in affected calves. Interference with fluoride absorption from the gut might have played a role in reducing plasma fluoride concentrations [1]. Presence of Water soluble proteins in MO have a strong positive charge that easily attracts highly electronegative fluoride ions which helps in forming flocculants [29]. Furthermore, the presence of tannins, fibers and high concentration of minerals in Moringa like calcium, aluminum, phosphorus, manganese, potassium, copper, and iron are reported to form insoluble complexes with fluoride in the gut [30]. Mean haematological parameters were significantly reduced in flourosis affected rats and reversed to normal in MO supplemented flourosis affected rats like Hb% and RBC. Mean haematological parameters values were not reduced in MO supplemented fluoride group as compared to group who were not supplemented with MO.No statistical significance was seen in group III & IV which shows that when fluoride was supplemented with MO toxic effects were reduced and findings were similar to group IV where the rats were fed only with MO.

## Limitations:

This study would have been better with prolonged duration & with more number of rats in each group. In future, studies should be continued on easily available plants for reducing the fluoride levels with greater duration to observe effect on haematological parameters other than HB & RBC.

## Conclusion:

Data shows that MO leaves powder is effective in reducing the plasma fluoride content. It also helps in improving the Hb % & RBC count in fluorosis affected rats. Thus, the usage of local and easily available plant products like MO in reducing fluoride levels & improved haematological effects due to fluorosis is reported.
